# The expression of respiratory tract virus in pediatric glomerular disease: a retrospective study of 45 renal biopsy in China

**DOI:** 10.1186/s12882-023-03083-8

**Published:** 2023-02-16

**Authors:** Li Lin, Lu Li, Yao Cao, Xin Peng, Yi Wu, Ping Yu, LiQun Dong

**Affiliations:** 1 Department of Pediatrics, West China Second University Hospital, Sichuan University, No. 20, Section 3, South Renmin Road, Chengdu, 610041 Sichuan Province China; 2 National Center for Birth Defect Monitoring, West China Second University Hospital, Sichuan University, No. 17, Section 3, South Renmin Road, Chengdu, 610041 Sichuan Province China; 3 Key Laboratory of Birth Defects and Related Diseases of Women and Children (Sichuan University), Ministry of Education, No. 17, Section 3, South Renmin Road, Chengdu, 610041 Sichuan Province China

**Keywords:** Pediatric glomerular diseases, Renal biopsy, Respiratory syncytial virus, Respiratory tract virus, RSV subtypes

## Abstract

**Background:**

More attention has been put on the relationship between pediatric glomerular disease and respiratory tract virus infection. Children with glomerular illness, however, are uncommonly found to have biopsy-proven pathological evidence of viral infection. The purpose of this study is to determine whether and what kind of respiratory viruses are found in renal biopsy from glomerular disorders.

**Methods:**

We used a multiplex PCR to identify a wide range of respiratory tract viruses in the renal biopsy samples (*n* = 45) from children with glomerular disorders and a specific PCR to verify their expression.

**Results:**

These case series included 45 of 47 renal biopsy specimens, with 37.8% of male and 62.2% of female patients. Indications for a kidney biopsy were present in all of the individuals. In 80% of the samples, respiratory syncytial virus was discovered. Following that, the RSV subtypes in several pediatric renal disorders were found. There were 16 RSVA positives, 5 RSVB positives, and 15 RSVA/B positives, accounting for 44.4%, 13.9%, and 41.7%, respectively. Nephrotic syndrome samples made up 62.5% of RSVA positive specimens. The RSVA/B-positive was detected in all pathological histological types.

**Conclusions:**

Patients with glomerular disease exhibit respiratory tract viral expression in the renal tissues, especially respiratory syncytial virus. This research offers new information on the detection of respiratory tract viruses in renal tissue, which may facilitate the identification and treatment of pediatric glomerular diseases.

**Supplementary Information:**

The online version contains supplementary material available at 10.1186/s12882-023-03083-8.

## Introduction

Glomerular disease is a group of illnesses with increased morbidity and death, overwhelming China's healthcare system [1]. Due to the variety of clinical presentations and treatment responses, there was not many high-quality clinical research on glomerular disease [2]. The most common manifestation of glomerular disease in children is nephrotic syndrome (NS), which is characterized by proteinuria, hypoalbuminemia, edema, and hyperlipidemia [3]. The most typical reason for a kidney biopsy in children is thought to be minimal-change nephrotic syndrome (MCNS) [4].

There have been some studies associating enteroviruses, Epstein–Barr virus (EBV), and cytomegalovirus (CMV) to nephropathy [5–7]. Infection, especially respiratory tract infection, is believed to be one of the risk factors for the onset, relapse, exacerbations, and development of end-stage renal disease (ESRD) in both adults and children with NS [8–11]. Previous studies have demonstrated that the respiratory syncytial virus (RSV), the most common respiratory virus, and its antibody were found in the urine, serum, epithelial cells of the respiratory tract, and peripheral blood mononuclear cells (PBMC) of patients with steroid-responsive nephrotic syndrome [12, 13], and a small percentage of intranasal inoculation of RSV can cause nephropathy in rats [14]. However, to our knowledge, no publication has connected respiratory virus infection with pediatric glomerular diseases through biopsy-proven pathological evidence.

The purpose of this research is to determine whether and which types of viral infections are found in renal biopsies from patients with glomerular disease. This study aims to offer visible proof of respiratory virus infection in children with glomerular diseases.

## Methods

### Ethics statement

The study was approved by the Institutional Review Board/Ethics Committee affiliated with West China Second University Hospital, Sichuan University (2,020,111), and followed the guidelines of national/international/institutional or Declaration of Helsinki. The informed consent was obtained from all participants’ guardians.

### Patients and clinical specimens

From January 2010 to December 2017, we collected data and renal biopsy specimens from 47 patients with pediatric glomerular disorders at Sichuan University's West China Second University Hospital.

The main indication of kidney biopsy was: (1) Steroid-resistant nephrotic syndrome (SRNS). (2) Steroid-dependent nephrotic syndrome (SDNS)/ Frequent relapsing NS (FRNS). (3) Nephrotic syndrome with hematuria, renal impairment, or persistent hypertension. (4) NS under the age of one year or over the age of ten years. (5) Proteinuria and glomerular hematuria. (6) Systemic illness with an abnormal urinalysis. (7) Unknown aetiology of acute or chronic renal failure. Pathological renal biopsy specimens were collected and maintained at -80 °C for subsequent detection of respiratory viruses.

**Fig. 1:**
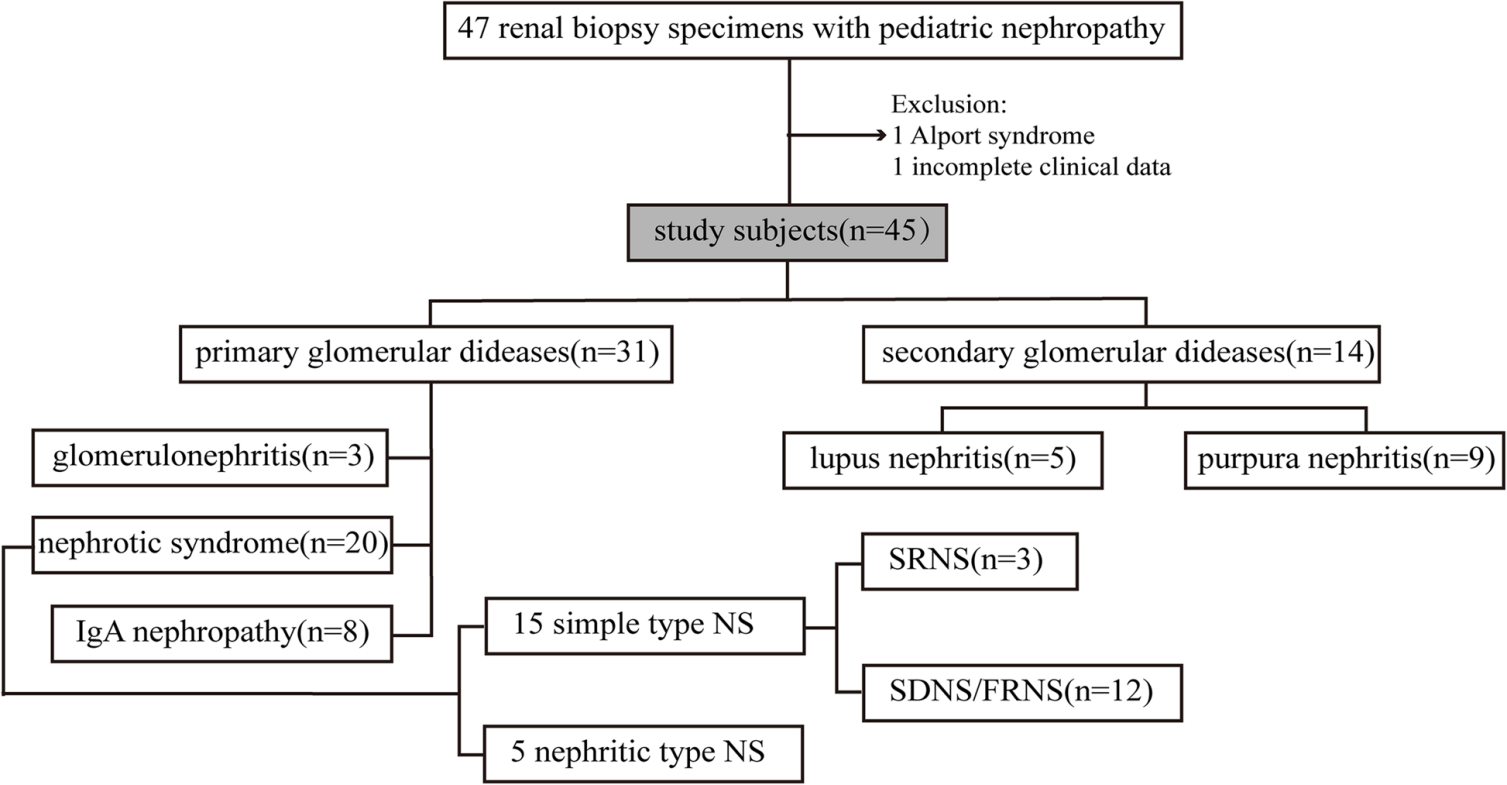
Flowchart of this study. NS, nephrotic syndrome; SRNS, steroid-resistant nephrotic syndrome; SDNS, steroid-dependent nephrotic syndrome; FRNS, frequent relapsing NS

Electronic medical records were reviewed retrospectively for clinical data. One subject was ruled out due to incomplete clinical data and 1 Alport syndrome were not included. The remaining 45 patients were admitted to our study (Fig. 1).

### Isolation of viral genomic RNA and cDNA synthesis

Viral genomic RNA was extracted from the patients’ renal biopsy specimens by using the QIAamp RNA extraction kit (Qiagen GmbH, Hilden, Germany), according to the protocol suggested by the manufacturer. Briefly, clinical samples were homogenized by vortexing for the 30 s, and 140 μl was used to extract viral genomic RNA. The RNA was eluted from the columns with 50 μl of elution buffer. The RNA was immediately stored at -80 °C for later detection. cDNA was synthesized by using MultiScribe reverse transcriptase and random hexamers (both from PE Applied Biosystems). The cDNA was stored at -80 °C before further use.

### Multiple PCR

Due to the diversity of the respiratory virus, we utilized multiplex nested PCR to test the respiratory tract virus in the meantime. A second (nested) amplification followed a one-tube RT-PCR. Seven sets of oligonucleotide primers were designed for mPCR and amplification of adenovirus, rhinovirus, influenza virus, parainfluenza virus, coronavirus, RSV, and GAPDH for internal reference, according to nucleotide sequences available from GenBank (shown in Table 1). Multiplex RT-PCR was performed by using a One-Step RT-PCR kit (Qiagen). Briefly, 5 μl of extracted RNA was added to a mixture composed of an enzyme mixture (CPG Inc., Lincoln Park, N.J.) and a mixture of the primers, each at a final concentration of 20 pmol. The optimized profile in the thermal cycler (PTC-100; MJ Research Watertown, Mass.) was 50 °C for 30 min and 95 °C for 15 min, followed by 35 amplification cycles (with each cycle consisting of denaturation at 94 °C for 45 s, annealing at 56 °C for 45 s, and synthesis at 72 °C for 1 min). Amplification was completed with a prolonged synthesis at 72 °C for 10 min.

**Table 1:** PCR Primers of the respiratory tract virus

**Virus**		**Primer sequence(5'-3')**	**Product(bp)**
Adenovirus	ADV-1	5'-CAGGACGCCTCGGAGTACCTGAG-3'	103
	ADV-2	5'-GGAGCCACGGTGGCGCC-3'	
Rhinovirus	RHV-1	5'-CGGACACCCAAAGTAG-3'	380
	RHV-2	5'-GCACTTCTGTTTCCCC-3'	
Parainfluenza Virus	PFV1-F	5'-GTTGTCAATGTCTTAATTCGTATCAATAATT-3'	108
	PFV1-R	5'-GTAGCCTACCTTCGGCAC-3'	
	PFV2-F	5'-GCATTTCCAATCTTCAGGACTATGA-3'	89
	PFV2-R	5'-ACCTCCTGGTATAGCAGTGACTGAAC-3'	
	PFV3-F	5'-AGTCATGTTCTCTAGCACTCCTAAATACA-3'	135
	PFV3-R	5'-ATTGAGCCATCATAATTGACAATATCAA-3'	
Influenza Virus	INFA-F	5'-GGACTGCAGCGTAGACGCTT-3'	188
	INFA-R	5'-CATCCTGTTGTATATGAGGCCCAT-3'	
	INFB-F	5'-AAGTACGGTGGATTAAATAAAAGCAA-3'	169
	INFB-R	5'-CCAGCAATAGCTCCGAAGAAA-3'	
Coronavirus	CV229E-F	5'-TGGCCCCATTAAAAATGTGT-3'	575
	CV229E-R	5'-CCTGAACACCTGAAGCAAT-3'	
	CVOc43-F	5'-GGCTTATGTGGCCCCTTACT-3'	334
	CVOc43-R	5'-GGCAAATCTGCCCAAGAATA-3'	
	CVNL63-F	5'-CTGTTACTTTGGCTTTAAAGAACTTAGG-3'	314
	CVNL63-R	5'-CTCACTATCAAAGAATAACGCAGCCTG-3'	
	CVHKU1-F	5'-ACCAATCTGAGCGAAATTACCAAAC-3'	443
	CVHKU1-R	5'-CGGAAACCTAGTAGGGATAGCTT-3'	
Respiratory Syncytial Virus	RSVAB-F	5'-GGAAACATACGTGAACAAGCTTCA-3'	80
	RSVA-R	5'-CATCGTCTTTTTCTAAGACATTGTATTGA-3'	
	RSVB-R	5'-TCATCATCTTTTCTAGAACATTGTACTGA-3'	81
GAPDH	GAPDH-F	5'-CGCTCTCTGCTCCTCCTGT-3'	81
	GAPDH-R	5'-CCATGGTGTCTGAGCGATGT-3'	

### Specific PCR with gel detection

Confirmatory tests were run to verify the initial results with specific PCR. Nested PCR was performed following multiplex PCR on specimens suspected of having RSV or another virus. For each nested PCR, a 0.1 μM concentration of each primer was used, 2 μM of first-run mPCR product was added, and an annealing temperature of 50℃ was used. Amplified products were electrophoretically separated on 2% agarose gels to differentiate virus-specific bands. The gels were then stained with ethidium bromide and visualized under UV light.

### Statistical analysis

The data management and statistical analysis were performed with GraphPad Prism 8 software (GraphPad Software, La Jolla, Calif).

## Results

### Baseline characteristics

This study included a total of 45 of 47 children with glomerular disease, with 17 (37.8%) males and 28 (62.2%) girls. Twenty patients had nephrotic syndrome, eight had primary IgA nephropathy, three had glomerulonephritis, five had lupus nephritis, and nine had purpura nephritis. NS was classified into two categories based on the clinical classification of glomerular disorders: simple type (15 patients) and nephritic type (5 patients). NS was categorized as nephritic type NS if at least one of the following criteria was met: 1) glomerular hematuria, 2) renal impairment, 3) chronic hypertension, and 4) low serum complement. Based on hormone sensitivity among the 15 patients with simple type NS, three children had SRNS and twelve children had SDNS/FRNS. A percutaneous kidney biopsy was performed on all 45 individuals since it was indicated. The average age at the time of kidney biopsy was 9.9 years old, ranging from 1.2-year-old to 18.3-year-old. Their median age at the commencement of the disease was 8.7 (range 1 to 17.7 years) years old. Twenty individuals suffered from respiratory tract infection when they had a kidney biopsy. Table 2 provided a description of the patients' clinical characteristics.

**Table 2:** The clinical features of patients with pediatric glomerular disease

	NS (*n* = 20)			IgA nephropathy (*n* = 8)	Glomerulonephritis (*n* = 3)	Secondary glomerular disease (*n* = 14)		Reference value
	SRNS(*n* = 3)	SDNS/FRNS(*n* = 12)	Nephritic type NS(*n* = 5)			Lupus nephritis(*n* = 5)	Purpura nephritis(*n* = 9)	
Gender								
Male (n)	1	6	1	5	0	0	4	··
Female (n)	2	6	4	3	3	5	5	··
The median age at the time of kidney biopsy (year)	4.4	11.2	9.8	6.9	11.1	12.5	10.8	··
The onset age at manifestation (year)	4.3	8.8	9.6	6.7	10.7	11.2	9.4	··
Respiratory tract infection at the time of biopsy(n)	3	8	2	3	3	0	1	··
Laboratory finding								
White blood cell (10^9/L)	13.8	10.1	12.1	8.9	9.5	7.9	9.9	3.6–9.7
Lymphocyte count (10^9/L)	5.3	3.6	3.7	2.9	2.1	2.0	2.0	1.22–4.16
Hemoglobin (g/L)	144.7	141.8	119.4	121.1	122.3	100.8	119.7	110–146
Platelet (10^9/L)	471	305.2	252	233.5	264	220.2	299	100–450
C-reactive protein(CRP, mg/L)	0.6	0.7	20.4	8.6	23.3	0.1	8.3	0–8
Antistreptolycin o (ASO, IU/ml)	9	11.2	372.4	38.8	174.3	61	53.5	0–200
IgG (g/L)	1.7	2.9	6.3	7.4	8.5	11.4	6.3	6.5–16
IgA (g/L)	1.0	1.8	1.9	1.8	2.0	1.8	2.1	0.29–2.7
IgM (g/L)	1.9	2.2	2.0	1.5	1.7	1.3	1.6	0.5–2.6
Albumin(g/L)	23.3	22.7	28.6	38.3	30.3	20.8	33.6	35–50
Serum creatinine(umol/L)	26.7	47.7	45.5	61.6	308.3	73.2	42.7	62–106
Proteinuria	2 + (1/3 patients)	2 + (5/12 patients)	··	··	··	1 + (1/5 patients)	··	Negative
	3 + (1/3 patients)	3 + (4/12 patients)	2 + (2/5 patients)	2 + (2/8 patients)	2 + (1/3 patients)	2 + (1/5 patients)	2 + (6/9 patients)	··
	4 + (1/3 patients)	4 + (3/12 patients)	3 + (3/5 patients)	3 + (1/8 patients)	3 + (2/3 patients)	3 + (3/5 patients)	3 + (3/9 patients)	··
24-h urine protein(g)	1.3	3	2.7	0.82	··	2.9	3.6	< 0.14 g/24 h
Hematuria	0 patient	5 ~ 10RBC/HPF (2/12 patients)	1 + (1/5 patients)	1 + (2/8 patients)	··	5 ~ 10 RBC/HPF (2/5 patients)	0 ~ 2 RBC/HPF (1/9 patients)	0 ~ 3RBC/HPF
	··	··	2 + (1/5 patients)	2 + (3/8 patients)	··	2 + (1/5 patients)	2 + (1/9 patients)	··
	··	··	3 + (1/5 patients)	3 + (3/8 patients)	3 + (1/3 patients)	3 + (1/5 patients)	3 + (4/9patients)	··
	··	··	4 + (2/5 patients)	··	4 + (1/3 patients)	4 + (1/5 patients)	4 + (3/9 patients)	··
Treatment at the time of biopsy(n)								··
Prednisolone	3	12	4	2	2	4	8	··
Cyclosporine	0	0	0	0	0	0	0	··
Cyclophosphamide	0	1	0	0	0	2	2	··
Tacrolimus	0	0	0	0	0	0	1	··
Hemoperfusion	0	0	1	0	1	0	1	··
Treatment after biopsy(n)								··
Prednisolone	3	12	5	5	2	5	9	··
Cyclosporine	0	0	0	0	0	0	0	··
Cyclophosphamide	2	1	2	1	0	2	5	··
Tacrolimus	0	4	0	0	0	0	3	··
Mycophenolate mofetil (MMF)	0	1	0	0	0	2	0	··
Hemoperfusion	0	0	0	1	1	1	3	··
Histopathology	MCD	MCD, FSGS, MN	MsPGN, CsGN, IgA nephropathy, MCD	MsPGN, IgA nephropathy, CsGN, MCD	MCD, IgA nephropathy	Lupus nephritis, FSGS	Purpura nephritis, MsPGN	··
Others	··	1 patient had dwarfism; 1patient had hepatitis; 1 patient suffered from attention deficit hyperactivity disorder	1 patient had hypertensive encephalophy	··	··	1 patient had hypertensive encephalophy; 1 patient died of respiratory failure	1 patient had hypertensive encephalophy	··

The definition of urine dipstick test about proteinuria: 1 + corresponds to less than 0.5 g/d of total protein in the urine; 2 + corresponds to 0.5 ~ 1 g/d of total protein in the urine; 3 + corresponds to 1 ~ 2 g/d of total protein in the urine; 4 + corresponds to greater than 2 g/d of total protein in the urine. The definition of urine dipstick test about hematuria: 1 + corresponds to 10-15RBC/high-power field (HPF) in the urine; 2 + corresponds to 15-30RBC/HPF in the urine; 3 + corresponds to greater than 30RBC/HPF but not in all field of microscope in the urine; 4 + corresponds to greater than 30RBC/HPF and in all field of microscope in the urine

*NS* Nephrotic syndrome, *SRNS* Steroid-resistant nephrotic syndrome, *SDNS* Steroid-dependent nephrotic syndrome, *FRNS* Frequent relapsing NS, *MCD* Minimal change disease, *FSGS* Focal segmental glomerulosclerosi, *MN* Membrane nephrosis, *MsPGN* Mesangial proliferative glomerylonephritis, *CsGN* Crescentic glomerulonephritis

### Respiratory virus infection was detected in renal biopsies

**Fig. 2:**
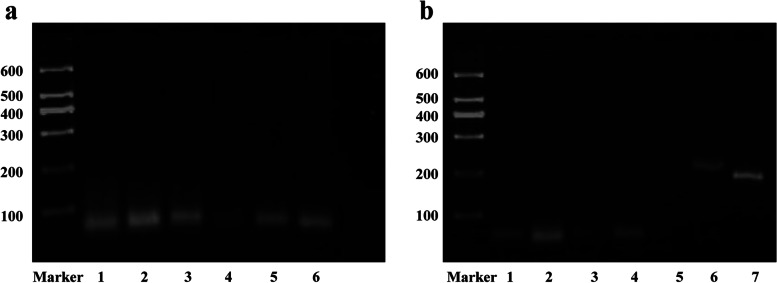
Respiratory virus expression in renal biopsy tissues. **a** The presence of RSVA in renal biopsy tissues was identified. The image showed that RSVA expression was present in lanes 1–3 and lanes 5–6. **b** RSVB and INFA/B was detected in renal biopsy tissues. The RSVB was expressed in lanes 1–2 and 4, INFA in lanes 6, and INFB in lanes 7. Viruses that are uncommon are not included

We utilized specific primers to identify the expression of adenovirus, rhinovirus, influenza virus, parainfluenza virus, coronavirus, and RSV in the samples. The result was shown on electrophoresis image. The expression of respiratory syncytial virus was detected in 36 (80%) and influenza virus was detected in 2 (4.4%) of the 45 kidney samples with pediatric glomerular disease. No common respiratory virus expression had been found in the rest of the sample (Fig. 2).

### Type of respiratory virus identified in pediatric glomerular disease renal biopsies

**Fig. 3:**
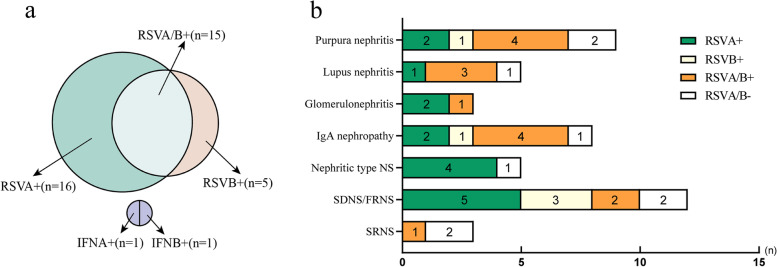
Respiratory virus subtype in pediatric glomerular disease renal biopsies. **a** Graphical representation of the classification of respiratory virus subtypes. **b** Various glomerular diseases and respiratory virus subtype

RSV was a single-stranded pneumovirus that belonged to the Pneumoviridae family [15]. RSV contained a negative-sense RNA genome that was separated into two genetic subtypes, RSV subgroups A (RSVA) and B (RSVB), based on antigenic and genetic diversity [16]. In 45 children's renal biopsies with glomerular disease, we found 36 RSV positive specimens, including 16 RSVA positive, 5 RSVB positive, and 15 RSVA/B positive specimens, accounting for 44.4%, 13.9%, and 41.7%, respectively (shown in Fig. 3a and Table 3). Nephrotic syndrome involved 62.5% of the RSVA positive samples, comprising four nephritic type NS and five SDNS/FRNS. Five RSVB specimens were identified in kidney biopsies, three of which were SDNS/FRNS (60%). In addition, the positive detection rate of RSVA/B in the Henoch-Schonlein purpura (HSP) nephritis, lupus nephritis, and IgAN specimens was 26.7% (*n* = 4), 20% (*n* = 3), and 26.7% (*n* = 4), respectively (Fig. 3b). Minimal change disease (MCD) was the most common cause of pediatric glomerular diseases as displayed in Table 3. A half of the RSVA-positive samples were histopathologically diagnosed with MCD. Only MCD, HSP nephritis and mesangial proliferative glomerylonephritis (MsPGN) were detected positive for the presence of RSVB, of which MCD accounted for 60%. The RSVA/B + was detected in all pathological histological forms.

**Table 3:** Respiratory syncytial virus subtype was detected in different renal biopsies

	n	MCD	HSPN	MsPGN	IgAN	LN	FSGS	CGN	MN
n	36	14	5	6	4	3	2	1	1
RSVA +	16	8 (50.0%)	1 (6.3%)	3 (18.8%)	2 (12.5%)	1 (6.3%)	1 (6.3%)	0	0
RSVB +	5	3 (60%)	1 (20%)	1 (20%)	0	0	0	0	0
RSVA/B +	15	3 (20%)	3 (20%)	2 (13.3%)	2 (13.3%)	2 (13.3%)	1 (6.7%)	1 (6.7%)	1 (6.7%)

*NS* Nephrotic syndrome, *SRNS* Steroid-resistant nephrotic syndrome, *SDNS* Steroid-dependent nephrotic syndrome, *FRNS* Frequent relapsing NS, *MCD* Minimal change disease, *FSGS* Focal segmental glomerulosclerosis, *MN* Membrane nephrosis, *MsPGN* Mesangial proliferative glomerylonephritis, *CsGN* Crescentic glomerulonephritis, *LN* Lupus nephritis, *HSPN* Henoch-Schonlein purpura nephritis, *IgAN* IgA nephropathy

The RSVA/B positive had nearly similar amounts of males and females. We did, however, find 12 females and 4 males with RSVA positivity. 4 out of 5 male kidney samples tested positive for RSVB.

In conclusion, renal tissues of children with glomerular disease were shown to include respiratory viruses, particularly RSV. Then, we identified RSV subtypes in various kidney biopsy specimens.

## Discussion

We retrospectively analyze renal biopsies from 45 pediatric patients with glomerular disease, over the course of seven years, in a tertiary care pediatric hospital in southwestern China. Kidney biopsy is regarded as the "gold standard" in the diagnosis of glomerular disorders. Based on their clinical profiles and the effectiveness of their treatments, these people need and successfully undergo a renal biopsy. The range of biopsy-verified pediatric glomerular disorders in China were characterized by Nie and Zheng [17, 18]. The most frequent reason for our patient's kidney biopsy (44.4%, 20/45) is nephrotic syndrome, which is consistent with their findings. Nevertheless, no study has linked respiratory viruses to glomerular disease in kidney biopsies. The purpose of this study is to identify the presence and kinds of respiratory tract virus expression in renal tissues in order to give pathological evidence of respiratory tract virus infection in pediatric glomerular disease.

Heterogeneity is one of the factors that contributed to the lack of high-quality clinical trials in primary glomerular disease [2]. In this case, our research may be of poor quality. To minimize the negative impact of homogeneous scarcity, we separate those individuals into groups depending on clinical symptoms and steroid hormone sensitivity. There are two categories for patients with glomerular disease: 1) primary disease group: nephrotic syndrome (SRNS, SDNS/FRNS and nephritic type NS), primary IgA nephropathy and glomerulonephritis; 2) secondary disease group: lupus nephritis and purpura nephritis.

Previous study has shown that bacteria, viruses, fungi, and mycobacteria all play a significant role in renal disease [19]. Individuals with glomerular disease are more prone to infection with hepatitis B, herpesviruses, parvovirus B19, respiratory syncytial virus, influenza virus (Flu), parainfluenza, enterovirus, and COVID-19 [20, 21]. According to our knowledge, respiratory virus infections play a prominent part in the initiation, aggravation, and recurrence of childhood NS and primary IgA nephropathy [10, 22]. According to the findings, when higher levels of corticosteroids are provided at the onset of a viral upper respiratory infection, relapses of NS are reduced [23]. We observe that respiratory virus is present in the majority of glomerular disease samples, implying that glomerular disease is related with viral infection of the respiratory tract. In previous research, respiratory tract viruses were detected in the airway epithelial cells, serum, urine, PBMC, and kidney tissue (only two specimens) of MCNS children in the active stage [12, 13, 24]. The virus in renal tissues, on the other hand, has attracted little study. In this study, multiplex PCR is utilized to directly detect the expression of possible respiratory viruses in pathologic renal biopsy specimens, resulting in visualized results indicating the presence of respiratory tract virus in pediatric glomerular disease. At the time of the biopsy, 45% of the subjects have an acute upper respiratory tract infection. We analyze that it has a moderate influence on the high percentage of respiratory tract virus positive (particularly RSV).

One of the study's advantages is the detection of RSV subgroups in numerous kidney biopsy specimens. RSVA is the most prevalent subtype in NS, while RSVA/B is the most common subtype in IgAN and secondary glomerular disease. Children with SDNS/FRNS are more prone to developing ESRD [25]. RSV was found in 83.3 percent of SDNS/FRNS tissues, implying that RSV infection may be a risk factor for SDNS/FRNS pathogenesis. We have learned that the type of renal histopathology is various in glomerular disease. MCD is the leading cause of pediatric glomerular disease in our study. RSVA/B-positive is found in a broad range of renal pathology specimens. There is also a low level of influenza virus expression, according to our data. It is unclear whether it has an impact on the incidence of pediatric nephropathy due to the small sample size.

Previous research has suggested that defective cell-mediated immunity may play an important role in the etiology of renal disease [26, 27]. RSV infection is a primary cause of proteinuria and renal dysfunction in the rat model of RSV reinfection. After a respiratory tract infection, primary T lymphocyte cells could impair cellular immune function and produce abnormal cytokines. RSV-induced anti-virus responses, in particular, can lead to uncontrolled cytokine production [28, 29]. According to a recent study, children with NS suffer renal damage and an inflammatory response [30]. We also found minor deterioration of renal function and low concentrations of immunoglobulin G (IgG) in glomerular disease patients, especially in NS, which is consistent with those results. However, we were unable to detect the expression of the apoptosis signal regulating kinase test, due to an insufficient testing facility in the early stages of the investigation.

It is important to note that this study has a number of limitations. First, even though this research is based on a retrospective case series, it is recommended to include some relevant mechanisms of RSV infection and pediatric glomerular disease. Furthermore, presenting the results of several control groups may help better reveal the association between viral infections and glomerular disease. Second, further investigation may focus on the connection between viral load and the severity of glomerular disease.

## Conclusion

In conclusion, our results support that respiratory tract viruses, with RSV being the most prevalent virus, can be detected in the renal tissues of individuals with glomerular disease. Our study adds to the knowledge of how important a role RSV infection may play in the pediatric glomerular disease.

## Supplementary Information

**Additional file 1.**

## Data Availability

All data generated or analysed during this study are included in this published article and its supplementary information files.

## Abbreviations

CMV: Cytomegalovirus
CsGN: Crescentic glomerulonephritis
EBV: Epstein–Barr virus
ESRD: End-stage renal disease
Flu: Influenza virus
FRNS: Frequent relapsing NS
FSGS: Focal segmental glomerulosclerosis
HSP: Henoch-Schonlein purpura
HSPN: Henoch-Schonlein purpura nephritis
IgAN: IgA nephropathy
IgG: Immunoglobulin G
LN: Lupus nephritis
MCD: Minimal change disease
MCNS: Minimal-change nephrotic syndrome
MN: Membrane nephrosis
MsPGN: Mesangial proliferative glomerylonephritis
NS: Nephrotic syndrome
PBMC: Peripheral blood mononuclear cells
RSV: Respiratory syncytial virus
RSVA: RSV subgroup A
RSVA/B: RSVA and B positive
RSVB: RSV subgroup B
SDNS: Steroid-dependent nephrotic syndrome
SRNS: Steroid-resistant nephrotic syndrome
