# Supplementary material for: The expression of respiratory tract virus in pediatric glomerular disease: a retrospective study of 45 renal biopsy in China
Source: BMC Nephrol. 2023 Feb 16;24:36. doi: 10.1186/s12882-023-03083-8 (PMC9931563; doi:10.1186/s12882-023-03083-8)

**The expression of respiratory tract virus in pediatric glomerular disease: a retrospective study of 45 renal biopsy in China**

**Authors:** Li Lin, Lu Li, Yao Cao, Xin Peng, Yi Wu, Ping Yu, LiQun Dong

The original images of Figure2 in this paper.

Figure 2a


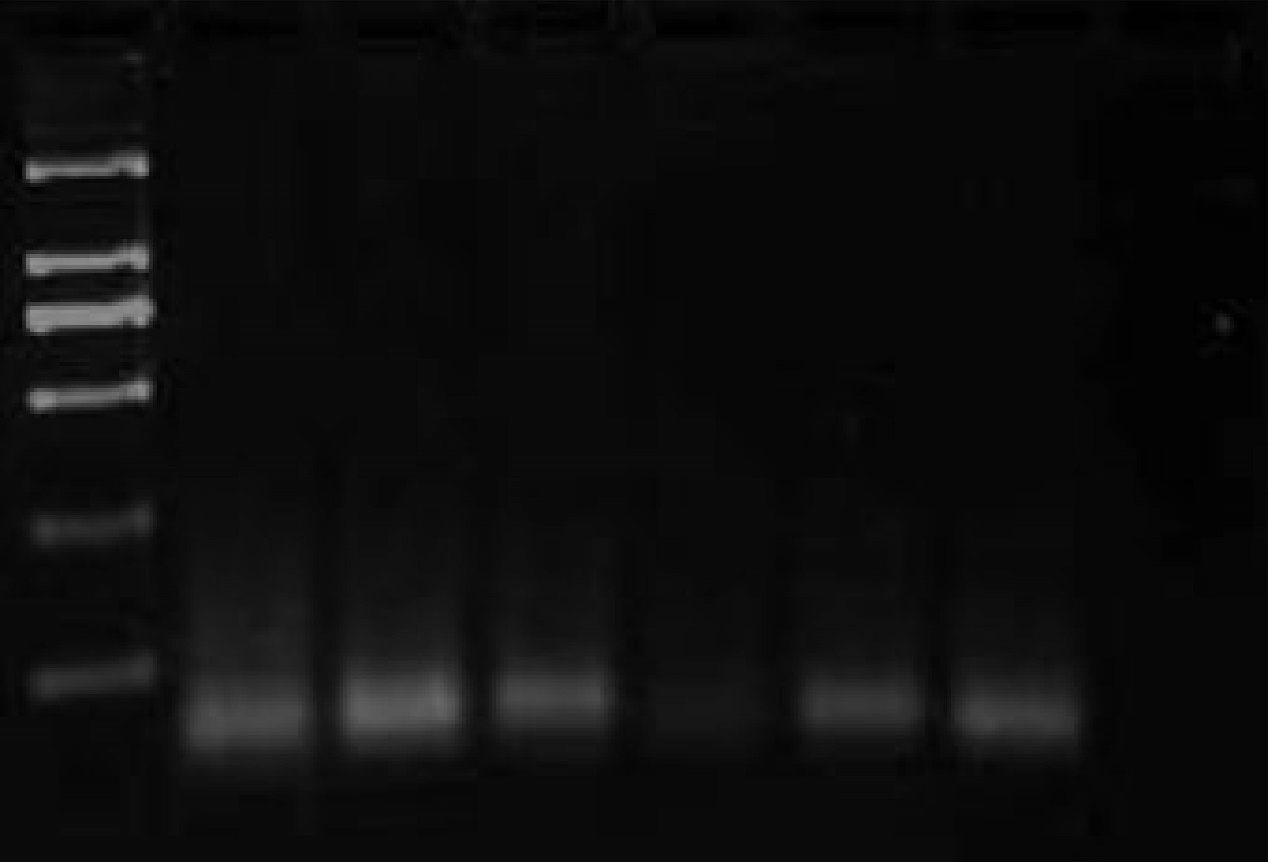


Figure 2b


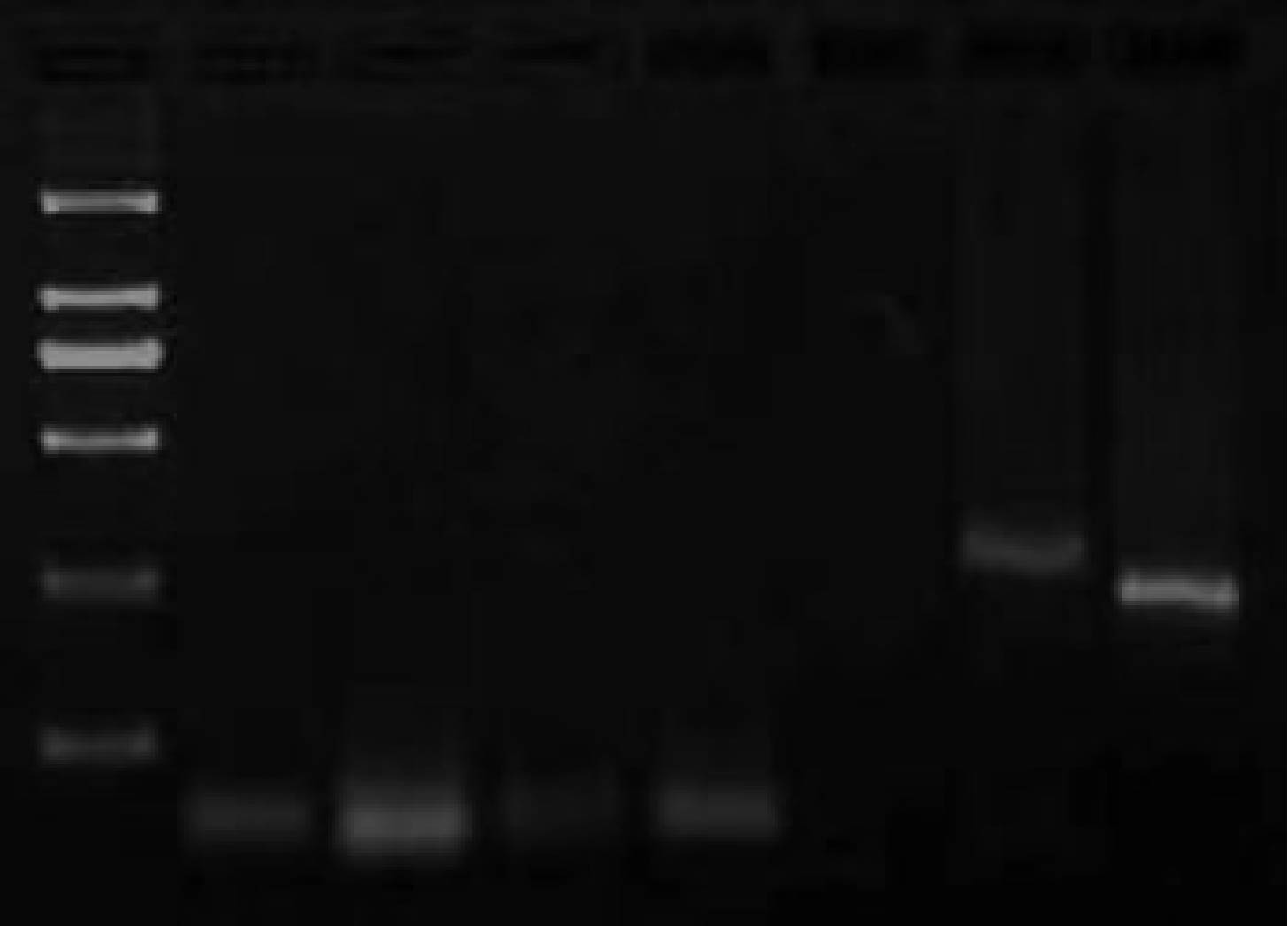

Supplement: Supplementary file 1 — Additional file 1. [file 12882_2023_3083_MOESM1_ESM.docx]
